# Association Between Acne Vulgaris and Body Mass Index in Adult Population: A Tertiary Hospital-Based Retrospective Study in Riyadh, Saudi Arabia

**DOI:** 10.7759/cureus.32867

**Published:** 2022-12-23

**Authors:** Yazeed Alowairdhi, Faisal Alrasheed, Faisal Alghubaywi, Muhannad Q Alqirnas, Waleed A Alajroush

**Affiliations:** 1 Medicine, King Saud Bin Abdulaziz University for Health Sciences, Riyadh, SAU; 2 Pediatric Dermatology, King Abdullah Specialized Children’s Hospital, Riyadh, SAU

**Keywords:** lifestyle, epidemiology, acne vulgaris, adults, kingdom of saudi arabia (ksa), body mass index: bmi, acne

## Abstract

Background/objectives: Acne vulgaris (AV) is the common form of acne, characterized by a polymorphic eruption of inflammatory non-papules, pustules, nodules, blackheads, and whiteheads. Acne was eighth among the top 10 disorders in terms of prevalence in 2010. The objective of this study was to see if there was any association between acne vulgaris and BMI in the young adult population.

Methods: This is a single-center retrospective study conducted at King Abdulaziz Medical City, Riyadh, Saudi Arabia. Adult patients diagnosed with acne vulgaris from January 2017 to June 2022 were enrolled. The estimated sample size was reached using consecutive, non-probability sampling.

Results: A total of 596 participants were selected as a match to the criteria of the research objectives. Of the participants, slightly more than half were males (52.5%) and the rest were females (47.5%). There was a nearly equal number of cases and controls, around 48.7% of the patients had acne while the rest did not. The majority were of the age group 18 to 19 years followed by 22 to 23 years (25.2%). A majority had a normal BMI of 18.5 to 24.9 (40.4%). A significant difference was found between the means of the BMI of the participants who had acne and those who did not (p<0.05).

Conclusion: No significant association was found between age group, gender, BMI, and acne. To fully comprehend how dietary factors affect the severity of acne, more studies are required.

## Introduction

Acne vulgaris (AV) is the common form of acne, which is defined as the formation of polymorphic eruption of inflammatory and non-inflammatory skin lesions, like papules, pustules, nodules, blackheads, and whiteheads. It has been rated eighth among the top 10 disorders in terms of prevalence in 2010 [1]. Also, it is one of the three most common skin diseases [2]. Acne vulgaris typically develops in the pilosebaceous unit. This unit is made up of the hair follicle and sebaceous gland, which secretes and produces sebum through the hair follicle's pores onto the skin's surface [3]. When the pilosebaceous unit's pore becomes plugged or irritated, AV lesions form [4]. Based on their size and appearance, the various forms of AV lesions (comedones, papules, pustules, nodules, and cysts) can be distinguished [3]. Due to their red and swollen look, papules, pustules, nodules, and cysts are commonly referred to as "inflammatory lesions," in contrast to comedones, which are frequently referred to as "non-inflammatory lesions" [4]. A person's AV severity can be roughly divided into three categories based on the lesions they have: mild, moderate, and severe. Milder AV only has non-inflammatory lesions, whereas more severe AV has both inflammatory and non-inflammatory lesions [4].

With a lifetime frequency of about 85%, AV is a very prevalent illness that predominantly affects adolescents [5]. Acne vulgaris can linger into adulthood; among women aged 20 to 29, the prevalence of AV was 50.9%, compared to 26.3% among those aged 40 to 49 [6].

Two-thirds of all dermatology office visits for AV are from female patients, and one-third of these visits are from women older than 25 years [7]. In Asians, AV typically affects women, with a male-to-female ratio of roughly 1/1.1:1.25. Most of these AV cases in women have a late onset [6,8]. In adolescent populations, moderate to severe AV is prevalent in 10% to 20% and is linked to psychosocial issues [9]. The idea that diet, lifestyle, and AV can be related is gaining more attention [10]. Increased sebum production in the skin due to obesity leads to severe AV. Body mass index has been mentioned in a few studies as a possible risk factor for the emergence of AV [11,12]. We recently studied the risk factors such as age, gender, and BMI that affect AV. The current study was intended to close the gap in the literature by analysing the relationship between age, gender, and BMI with AV severity.

## Materials and methods

This is a single-center retrospective study conducted at King Abdulaziz Medical City, Riyadh, Saudi Arabia. The study was approved by the ethics committee of King Abdullah International Medical Research Center (IRB NRC22/240/05). Adult patients diagnosed with AV from January 2017 to June 2022 were enrolled, totaling 290 patients. The control group comprised 306 healthy individuals of the same population, comparable in age and gender. The estimated sample size was reached using consecutive, non-probability sampling.

Data were acquired by searching electronic medical records. Study variables included age, gender, BMI, date of birth, and visit date. Data were analyzed using the Statistical Package for the Social Sciences (SPSS) version 23.0 (IBM Corp., Armonk, NY, USA). Frequency and percentage were produced for the categorical variables and mean and standard deviation (SD) for the quantitative variables. The chi-square test was used for the bivariate analysis of categorical data. Paired t-tests and ANOVA tests were used for the bivariate analysis of numerical data. For all statistical analyses, a p-value of less than 0.05 was considered significant.

## Results

Sociodemographic characteristics

**Figure 1:**
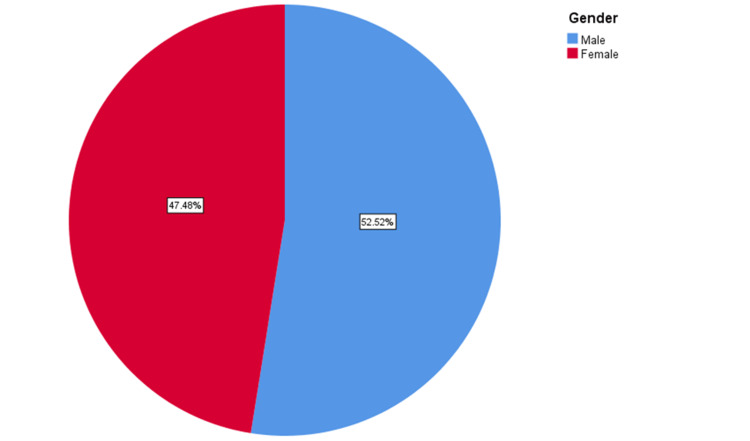
Gender-wise distribution of participants

A total of 596 participants were selected as a match to the criteria of the research objectives. Of the participants, 52.5% were males and the rest were females (47.5%) (Figure 1). 

There was a nearly equal number of cases and controls; around 48.7% of the patients had AV while the rest did not. Majority of the participants belonged to the 18 to 19 years age group followed by 22 to 23 years (25.2%), 20 to 21 years (25.0), and finally 24 to 25 years (19.3%). Interestingly, a majority had a normal BMI within 18.5 to 24.9 (40.4%) followed by those who were overweight (31.9%), obese class I (13.8%), underweight (7.7%), obese class II (3.5%), and obese class III (2.9%) (Table 1).

**Table 1:** Sociodemographic characteristic of the participants AV: Acne vulgaris

		Frequency	Percentage (%)
Gender	Male	313	52.5
	Female	283	47.5
AV vs Control	AV Patients	290	48.7
	Control	306	51.3
Age Group	18 to 19 Years	182	30.5
	20 to 21 Years	149	25.0
	22 to 23 Years	150	25.2
	24 to 25 Years	115	19.3
BMI Groups	Underweight (<18.5)	46	7.7
	Normal (18.5-24.9)	240	40.3
	Overweight (25-29.9)	190	31.9
	Obese Class I (30-34.9)	82	13.8
	Obese Class II (35-39.9)	21	3.5
	Obese Class III (>40)	17	2.9
Total		596	100.0

**Figure 2:**
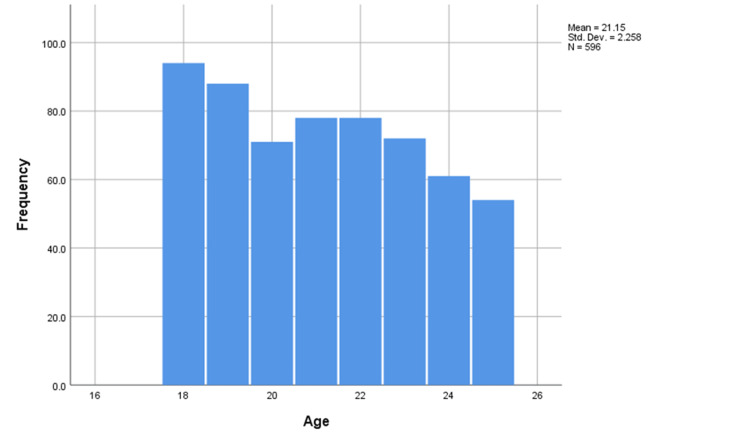
Histogram of ages of the participants Std.Dev: Standard deviation

**Figure 3:**
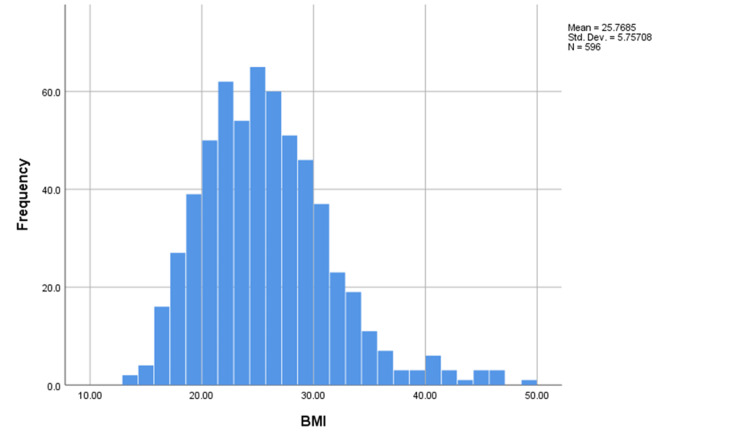
Histogram of BMI of the participants Std.Dev: Standard deviation

The mean age of the participants was 21.15 years, the median was 21 years and the mode was 18 years indicating a negatively skewed distribution of the variable (Figure 2). Interestingly, the mean and median BMI of the participants were 25.7 and 25.1, respectively (Figure 3).

Association of AV with age, gender, and BMI

The highest prevalence of AV was among the 24 to 25 years age group (56.5%), females (51.6%), and obese class II (66.7%) among their respective categories. The least prevalence of AV was among the 22 to 23 years age group (54.0%), males (54.0%), and normal BMI group (54.2%). However, no significant association was found between age, gender, BMI and the AV group and controls (Table 2).

**Table 2:** Distribution of cases and control with respect to the sociodemographic characteristics

			AV vs Control		Total	
			AV Patients	Control		
Age Group	18 to 19 Years	Count	85	97	182	χ2 = 3.610, df = 3, p=0.307
		Percentage (%)	46.7	53.3	100.0	
	20 to 21 Years	Count	71	78	149	
		Percentage (%)	47.7	52.3	100.0	
	22 to 23 Years	Count	69	81	150	
		Percentage (%)	46.0	54.0	100.0	
	24 to 25 Years	Count	65	50	115	
		Percentage (%)	56.5	43.5	100.0	
Gender	Male	Count	144	169	313	χ2 = 1.855, df = 1, p=0.173
		Percentage (%)	46.0	54.0	100.0	
	Female	Count	146	137	283	
		Percentage (%)	51.6	48.4	100.0	
BMI Groups	Underweight (<18.5)	Count	24	22	46	χ2 = 7.416, df = 1, p=0.192,
		Percentage (%)	52.2	47.8	100.0	
	Normal (18.5-24.9)	Count	110	130	240	
		Percentage (%)	45.8	54.2	100.0	
	Overweight (25-29.9)	Count	100	90	190	
		Percentage (%)	52.6	47.4	100.0	
	Obese Class I (30-34.9)	Count	47	35	82	
		Percentage (%)	57.3	42.7	100.0	
	Obese Class II (35-39.9)	Count	14	7	21	
		Percentage (%)	66.7	33.3	100.0	
	Obese Class III (>40)	Count	11	6	17	
		Percentage (%)	64.7	35.3	100.0	
Total		Count	306	290	596	
		Percentage (%)	51.3	48.7	100.0	

Interestingly, using an independent sample t-test, a significant difference was found between the means of the BMI of the participants who had AV and those who did not (p<0.05). The mean BMI of the cases was found significantly less than the mean BMI of the controls. However, no significant difference was found between the means of ages of those with and without AV (p=0.125) (Table 3).

**Table 3:** Comparison of means of BMI and age of cases and controls AV: Acne vulgaris, Std.: Standard

	AV vs Control	N	Mean	Std. Deviation	Std. Error Mean
BMI**	AV Patients	290	25.2381	5.29068	.31068
	Control	306	26.2713	6.13302	.35060
Age*	AV Patients	290	21.30	2.323	.136
	Control	306	21.02	2.190	.125
**Independent Samples t-test, t = -2.197, p<0.05, Significant * Independent Samples t-test, t = 1.535, p=0.125, Insignificant					

As evident in Table 4, using a binary logistic regression test, a significant association was found between having AV and the BMI of the participant before adjustment of the odds ratio (OR). It was found that for each unit rise in the BMI, the participants were almost 3% less likely to have AV (crude odds ratio (COR)=0.969, p<0.05). Non-adjusted or COR predicts the outcome only on the basis of one independent variable/predictor at a time. When looking at a logistic regression model with only BMI as a predictor of AV, a significant association was found. However, this was before the adjustment of other possible confounders. In the adjusted OR model, the outcome of AV was predicted on the basis of BMI, age, and gender of the participant where none of the predictors were found to be significantly associated with the outcome

**Table 4:** Association of AV with the gender, age, and BMI of the participants OR: Odds ratio

		Unadjusted OR	p-value	Adjusted OR	p-value
Gender	Female	1.251	0.173	1.253	0.176
	Male	Ref	Ref	Ref	Ref
Age		1.057	0.125	1.057	0.137
BMI		0.969	0.029*	0.972	0.052
*p<0.05, Significant					

## Discussion

The prevalence of obesity has risen globally [13]. A number of metabolic diseases, including diabetes, metabolic syndrome, and polycystic ovarian syndrome (PCOS), are associated with obesity [14,15]. Acne vulgaris may worsen as a result of the hyperandrogenism that obesity fosters within the body. According to Alan et al., having a higher BMI causes hyperandrogenism and worsening AV [16]. For the same reason, several contraceptive tablets that block androgen activity are recommended in the updated therapy guidelines for AV [17]. Cibula et al., on the contrary, state there is no relationship between AV and androgen overproduction [18]. Numerous researchers have looked at the association between obesity and AV, and the majority of them have found that having a BMI >25 kg/m2 is a substantial risk factor for AV [19].

Studies on AV in young adults and teenagers show that having a high BMI can make AV worse [20]. According to these findings, having a low BMI protects against AV [21]. Estrogens are known to reduce sebum production and to counteract androgens’ actions on the sebaceous glands on the skin, therefore, owning a protective role against AV [22-23]. Obesity and intra-abdominal fat have been proven to have a positive relation to the levels of estradiol, and an inverse relation to the total testosterone concentration [24,25]. Other studies reported that obesity decreased the activity of 5-a reductase-II, which transforms testosterone to the more physiologically active dihydrotestosterone [26,27].

One study found a significant correlation between higher BMI and higher insulin-like growth factor expression, which in turn enhanced AV severity, highlighting the value of nutritional treatment for AV [28]. According to Lu (&) Hsu, children with comedonal AV have lower BMIs than those with inflammatory AV [29]. Losing weight will lessen the amount of inflammatory bacteria that cause lipolysis on the fascial skin, enhancing AV treatment regimens [30]. However, other studies asserted that there was no connection between BMI and AV that developed after adolescence [29].

In our study, the highest prevalence of AV was among obese class II (66.7%). Interestingly a significant difference was found between the means of the BMI of the participants who had AV and those who did not (p<0.05). The mean BMI of the AV group was found significantly less than the mean BMI of the controls. It was found that for each unit rise in the BMI, the participants were almost 3% less likely to have AV (COR=0.969, p<0.05) (as seen above in Table 4).

Numerous studies have shown that the development of AV frequently coincides with the start of puberty when sebum production rises [5]. *Propionibacterium acnes*, a bacterial species implicated in the inflammatory processes in AV and the development of inflammatory AV lesions, which are often linked to more severe AV, thrive in environments with high levels of sebum [5,31]. As a result, the prevalence of AV rises with age, peaking in adolescence and declining significantly in prepubescent children [5]. When a person reaches late adolescence or early adulthood, the prevalence of AV declines with age [32]. The earlier onset of puberty in females compared to boys may be the cause of the higher AV prevalence in females at younger ages, according to Lynn et al. [2]. However, the studies included in this analysis produced conflicting findings, with just two revealing a higher likelihood of AV in females and three demonstrating a higher likelihood of AV in males. These findings can be the consequence of variations in the characteristics of the sampled population or the nation under investigation [33]. In our study, the highest prevalence of AV was among the age group 24 to 25 years (56.5%) and the least prevalence of AV was among the 22 to 23 years age group (54.0%).

In comparison to less than 5% of adult men, 22% of adult women have AV, according to The Journal of Clinical and Aesthetic Dermatology. Diet is a risk factor for AV in adult men and women [34]. Low consumption of fruits and vegetables is linked to the development of AV, however, eating fish helps prevent the condition, which is most likely because it contains omega-3 fatty acids [35]. In a study on AV-prone people in Leeds, eating dark chocolate was found to increase the number of comedones and inflammatory lesions [36]. Teenage AV has been linked to high glycemic load diets and dairy products such as skimmed milk and chocolate [37].

These diets enhance lipogenesis, sebum production, and keratinocyte proliferation, which exacerbates AV [38]. Consuming bread and cake has not been linked to an increased risk of AV. Additionally, it has not been discovered that greasy and spicy foods cause AV [37].

Premenstrual flares, or an increase in the quantity of papules and pustules one week prior to menses, affect 24% to 78% of women [39]. Di Landro et al. did not discover a link between premenstrual flares and the severity of AV in their study of young people [35]. Premenstrual flares are documented in most studies of adult women, although they are not correlated with the severity of AV [40]. In our study, the highest prevalence of AV was also among females (51.6%), and the least prevalence of AV was among males (54.0%).

This study should be interpreted in light of its strength and limitations. One limitation is the retrospective nature of this study which might weaken this study. Another limitation is that this study was conducted in only one center and did not include all age groups. Lastly, this study took a sample of the whole population which might increase the bias probabilities. Its strength is that it was conducted in a tertiary hospital with a wide variety of patient backgrounds and included enough samples to represent the whole population.

## Conclusions

No significant association was found between age group, gender, BMI, and AV. To fully comprehend how age, gender, and BMI affect the severity of AV, prospective multicentre studies are required to reach a definitive consensus. The results of this study can also serve as a roadmap for future studies on risk factors in the pursuit of a deeper comprehension of the pathophysiology of AV and the creation of more effective treatments.
